# BCAS2 regulates granulosa cell survival by participating in mRNA alternative splicing

**DOI:** 10.1186/s13048-023-01187-1

**Published:** 2023-05-29

**Authors:** Xiaohong Yao, Chaofan Wang, Longjie Sun, Lu Yan, Xuexue Chen, Zheng Lv, Xiaomei Xie, Shuang Tian, Wenbo liu, Lei Li, Hua Zhang, Jiali Liu

**Affiliations:** 1 State Key Laboratory of Animal Biotech Breeding, College of Biological Sciences, China Agricultural University, Beijing, 100193 China; 2 Department of Obstetrics and Gynecology, Center for Reproductive Medicine, Guangdong Provincial Key Laboratory of Major Obstetric Diseases, The Third Affiliated Hospital of Guangzhou Medical University, Guangzhou, China; 3 State Key Laboratory of Stem Cell and Reproductive Biology, Institute of Zoology, Chinese Academy of Sciences, Beijing, 100101 China

**Keywords:** BCAS2, Alternative splicing, Cell cycle, PRP19 complex, DNA damage

## Abstract

**Background:**

Granulosa cell proliferation and differentiation are essential for follicle development. Breast cancer amplified sequence 2 (BCAS2) is necessary for spermatogenesis, oocyte development, and maintaining the genome integrity of early embryos in mice. However, the function of BCAS2 in granulosa cells is still unknown.

**Results:**

We show that conditional disruption of *Bcas2* in granulosa cells caused follicle development failure; the ratio of the positive cells of the cell proliferation markers PCNA and Ki67 were unchanged in granulosa cells. Specific deletion of *Bcas2* caused a decrease in the BrdU-positive cell ratio, cell cycle arrest, DNA damage, and an increase in apoptosis in granulosa cells, and RPA1 was abnormally stained in granulosa cells. RNA-seq results revealed that knockout of *Bcas2* results in unusual expression of cellular senescence genes. BCAS2 participated in the PRP19 complex to mediate alternative splicing (AS) of *E2f3* and *Flt3l* mRNA to inhibit the cell cycle. Knockout of *Bcas2* resulted in a significant decrease in the ratio of BrdU-positive cells in the human granulosa-like tumour (KGN) cell line.

**Conclusions:**

Our results suggest that BCAS2 may influence the proliferation and survival of granulosa cells through regulating pre-mRNA splicing of *E2f3* and *Flt3l* by forming the splicing complex with CDC5L and PRP19.

**Supplementary Information:**

The online version contains supplementary material available at 10.1186/s13048-023-01187-1.

## Introduction

The ovary is the female reproductive organ, consisting of oocytes and somatic cells (granulosa cells, theca cells, endothelial cells, supporting connective tissue) [1]. Primary ovarian insufficiency (POI) or premature ovarian failure (POF) is a common cause of female infertility [2–4]. POF is the terminal state of POI [5], and the state and quality of granulosa cells in the ovary are crucial factors affecting POF [6, 7]. Proliferation and differentiation of granulosa cells are essential for follicle maturation and ovulation.

Abnormal apoptosis and degeneration of granulosa cells lead to follicular atresia [8, 9]. Since granulosa cell dysfunction is associated with POI, determining the physiological function of granulosa cells will provide important insights into the pathogenesis of POI.

Previous studies have reported that AS plays an essential role in reproduction. In granulosa cells, the androgen receptor insertion and deletion isoforms of AS variants disrupted follicle growth and androgen metabolism, resulting in polycystic ovary syndrome (PCOS), which is a common disease that results in infertility [10]. In male germ cells, deleting the MORF-related gene on the chromosome resulted in abnormal *Tnp2* pre-mRNA splicing and arrested spermatogenesis [11]. In oocytes, deletion of epithelial splicing regulatory protein 1 interrupted pre-mRNA splicing and resulted in perturbed spindle organization, chromosome alignment, metaphase-to-anaphase transformation, and female infertility [12].

BCAS2 was first found to be a highly expressed gene in human breast cancers [13] and involved in pre-mRNA AS [14–18] and DNA damage repair [15, 19–22]. Replication protein A (RPA) is a heterotrimeric single-stranded DNA-binding complex composed of 70 kDa (RPA1), 32 kDa (RPA2), and 14 kDa (RPA3) subunits and is essential for DNA replication, recombination, and repair in eukaryotes [23, 24]. The PSO4 complex (PSO4/PRP19/SNEV, CDC5L, PLRG1, and BCAS2/SPF27) modulates the DNA damage response via its interaction with RPA. The ability of BCAS2 to bind RPA1 and the E3 ligase activity of PSO4 are both required for the efficient accumulation of ATRIP at DNA damage sites and RPA2 phosphorylation [21]. Conditional disruption of BCAS2 in germ cells led to mouse infertility. Specific disruption of *Bcas2* in male germ cells affected mouse spermatogenesis and male fertility by regulating AS, which decreases the full-length formation of deleted azoospermia-like (*Dazl*) [25]. *Vasa-Cre* mediated deletion of *Bcas2* caused poor oocyte quality, abnormal oogenesis, and follicular development, possibly by regulating the AS of functional genes through the PRP19 complex [26]. Maternal depletion of *Bcas2* by *Zp3-Cre* mice destroyed the genomic integrity of early embryos and resulted in female infertility [22]. However, the function of BCAS2 in granulosa cells is still unknown.

In this study, we used a *Foxl2-CreER*^*T*2^ mouse model to knock out *Bcas2* in granulosa cells [27]. The results showed that lacking of BCAS2 in granulosa cells results in the loss of secondary follicles. Granulosa cells undergo DNA damage and apoptosis. RNA-seq results revealed that BCAS2 regulates the cell cycle by participating in *E2f3* and *Flt3l* mRNA splicing through the PRP19 complex, and knocking down *BCAS2* in the KGN cell line significantly reduced proliferation. Thus, BCAS2 in granulosa cells is essential for female fertility.

## Materials and methods

### Mice

All mice were housed in the animal facilities of China Agricultural University with free access to food and clear water. The temperature was between 22 and 26 °C, and the light conditions were 12 h (h) in light and 12 h in darkness. The *Bcas2*^*F/F*^; *Foxl2-CreER*^*T2*^ lines were initially derived on a mixed background (129/C57BL/6) but maintained on C57BL/6 mice. Pups were injected with 20 mg/kg tamoxifen (T5648; Sigma, St. Louis, MO, USA) at 1 day postpartum (dpp), 3 dpp, and 5 dpp to activate Cre recombinase. The cultivation, breeding, and experimental operation of the mice followed the guidelines of animal research at China Agricultural University.

## Identification of mouse genotypes

DNA was extracted from the mouse tail by the HotSHOT method, and PCR was used to distinguish different genotypes with specific primers. Taq Master Mix (2×) (Dye Plus) (P112-AA, Vazyme, Nanjing, China) was used for PCR. The common PCR conditions were as follows: 95 °C for 5 min; 36 cycles of 95 °C for 30 s, 58 °C for 30 s, and 72 °C for 40 s; 72 °C for 5 min; and holding at 4 °C. PCR products were seperated with 2% agarose. The size of the PCR product bands was used to distinguish wild-type (WT) from floxed (Flox). The WT size was 402 bp, and the Flox size was 522 bp. PCR primer sequences are shown in Supplementary Table 1 (Table S1).

## Ovary histological analysis and follicle counts

Ovaries were collected on different days, fixed in 4% paraformaldehyde (P6148; Sigma, St. Louis, MO, USA) overnight at 4 °C. Then, the ovaries were embedded in paraffin blocks, sectioned at a thickness of 5 μm, and stained with haematoxylin and eosin (H&E). The follicles were counted and classified in every fifth section at 7 dpp, 14 dpp, and 21 dpp as follows: one layer of squamous granulosa cells we called primordial follicles, one layer of cubic granulosa cells we called primary follicles, two or more layers of granulosa cells we called secondary follicles, follicles with a large cavity multilayer and multiple layers of granulosa cells we called antral follicles, granulosa cell abnormalities and apoptosis we called atretic follicle [28].

## Immunofluorescence

Sections of the ovary were dewaxed and microwaved with 0.01 M sodium citrate buffer for antigen retrieval (pH = 6.0). Nonspecific antigen was blocked with 10% normal goat serum (SP-9000; Zhongshan Golden Bridge Biotechnology, Beijing, China) at room temperature (RT) for 1 h. The primary antibodies (Table S2) were incubated at 4 °C overnight, washed with phosphate-buffered saline (PBS), incubated with diluted secondary antibodies (Table S2) at RT for 2 h, washed with PBS, and incubated with 4′,6-diamidino-2-phenylindole (DAPI) (10,236,276,001; Roche Applied Science, Basel, Switzerland) at RT for 10 min. Finally, the sections were treated with an antifade mounting medium (P0128S; Beyotime Biotechnology, Shanghai, China). A Nikon A1 laser scanning confocal microscope (A1, Nikon, Japan) was used for imaging immunofluorescent sections.

Cell immunofluorescence: The cells were fixed with precooled methanol for 20 min, washed with PBS 3 times, permeated with 0.2% Triton X-100 for 15 min, and blocked with 10% normal goat serum. Subsequent steps for immunofluorescence were performed as described above.

## Detection of proliferation by BrdU

Mice were injected with 100 mg/kg 5-bromo-2’-deoxyuridine (BrdU) (B5002, Sigma, St. Louis, MO, USA) at 7 dpp, 10 dpp, or 14 dpp. After 2 h, ovarian tissue was collected, fixed, embedded, sliced, microwaved with EDTA antigen retrieval solution (pH = 9.0), and washed with PBS. BrdU antibody was added to the tissue overnight at 4 °C, and then, the tissue was washed with PBS. Secondary antibody was added at RT for 2 h, the sample was washed with PBS, and DAPI was incubated at RT for 10 min. Then, the sample was washed with PBS. Mounting and imaging were performed as above.

## Apoptosis analysis by TUNEL

Apoptosis of the ovarian tissue was detected by One Step TUNEL Apoptosis Assay Kit (C1088; Beyotime Biotechnology, Shanghai, China) according to a standard protocol. We chose the largest section of the ovary and counted total granulosa cell number and apoptotic cell number in the section.

## Cell culture, cell transfection, and cell proliferation assays

The KGN cell line (Shanghai Binsui Biotechnology Co., Ltd.) was cultured in DMEM (HK2109.07; Huanke Technology, Beijing, China) containing 10% fetal bovine serum (S500; Newzerum, Ltd., Christchurch, New Zealand) and 1% penicillin and streptomycin at 37 ℃ in a 5% CO_2_ incubator. pMKO.1 GFP was a lentivirus expression vector and as a negative control. The sequences were as follows: sh-163, AAGAACTACCTGAGCTACCTG; sh-316, AATGACATTACTGCATGGCAA; sh-391, AATCTGGAACTAATGTCACAG. A total of 2.5 µg of shRNA was transfected into the KGN cell line using Lipo8000™ (C0533; Beyotime Biotechnology, Shanghai, China) according to the manufacturer’s instructions. After 72 h of cell culture, 20 ng/mL BrdU (5002-1G; Sigma, St. Louis, MO, USA) was added for 2 h.

### Western blot and coimmunoprecipitation (Co-IP)

The ovary was added to 50 µl of RIPA buffer (P0013B; Beyotime Biotechnology, Shanghai, China) and mashed with a grinding rod. Then, the samples were frozen and thawed five times with liquid nitrogen and centrifuged at 12,000 rpm at 4 °C for 30 min (min). After that, the supernatant was aspirated, and the protein concentration was measured with a BCA Protein Assay Kit (CW0014; Cwbiotech, Jiangsu, China). Total proteins were separated by 12% sodium dodecyl sulfate-polyacrylamide and transferred to polyvinylidene difluoride membranes (IPVH00010; Millipore, USA) by electricity (Bio-Rad, Hercules, USA). The membrane was blocked with 5% skim milk (P0216-300 g; Beyotime Biotechnology, Shanghai, China) at RT for 1 h. First, the membrane was incubated with BCAS2 antibody and GAPDH antibody overnight at 4 °C and then washed with 0.05 M Tris-buffered saline and 0.1% Tween-20 (TBST, pH 7.4) three times, incubated with secondary antibodies (Table S2) at RT for 2 h, and washed with TBST three times. After that, the membrane was detected with BeyoECL Plus (P0018S; Beyotime Biotechnology, Shanghai, China) and visualized by a Tanon 5200 chemiluminescent imaging system (Tanon, Shanghai, China).

For IP analysis, eight-day-old ovaries were solubilized in cell lysis buffer (P0013; Beyotime Biotechnology, Shanghai, China) and then centrifuged until the supernatant was retained. Rabbit IgG and BCAS2 antibodies were adsorbed by BeyoMag™ Protein A (P2102; Beyotime Biotechnology, Shanghai, China), and then, cell lysate was adsorbed by beads overnight at 4 °C. The beads were washed three times with TBST and boiled in 1× SDS loading buffer, and Western blot was performed as described above. Immunoblotting was performed with specific antibodies against BCAS2, PRP19, and CDC5L.

## qPCR, RT‒PCR, and RNA immunoprecipitation (RIP)

The total RNA of the ovary was extracted by TRIzol Reagent (9109; TaKaRa, Dalian, China), and the RNA concentration was measured by a NanoDrop (ND-2000, USA). One microgram of mRNA was transcribed into cDNA using M-MLV (M170A; Promega, USA). Quantitative PCR (qPCR) analysis was performed with Hieff UNICON® qPCR SYBR Green Master Mix (11198ES03; Yeasen, Shanghai, China) by an LightCycler® 96 (Roche, Switzerland). GAPDH was used as an internal reference. qPCR primer sequences are shown in Supplementary Table 3.

cDNA was used for common PCR with 2 × Taq Master Mix (Dye Plus). Primers used to determine mature mRNAs of *E2f3* and *Flt3l* were designed for RT‒PCR. Information on all primers is listed in Supplementary Table 4. RNA is extracted from ovarian tissue and then reversed into cDNA, using cDNA as a template. The common PCR conditions were as follows: 95 °C for 5 min; 36 cycles of 95 °C for 30 s, 58 °C for 30 s, and 72 °C for 40 s; 72 °C for 5 min; and holding at 4 °C. PCR products were seperated with 2% agarose. The RIP assay protocol was performed as described in a previous report [29].

## RNA-seq

Ovarian samples were collected at 8 dpp and sent to Novogene for RNA-seq analysis. Total RNA was isolated. The concentration was assessed with a Nanodrop (NanoDrop Technologies; Wilmington, DE, USA) and an Agilent 2100 (Agilent; Palo Alto, CA, USA). The quality was determined with an Agilent 2100, and purity was determined with an Agilent 2100 and agarose gel electrophoresis. The cDNA library was generated by mRNA using the NEBNext® Ultra™ RNA Library Prep Kit for Illumina®. The RNA-seq library was sequenced with an Illumina following sequencing. After quality control of the date, adapter reads, unknown reads, and low-quality reads were removed.

## AS analysis

The clean reads were used for analysis. rMATS software (version 3.2.5) was used for quantitative and differential analysis of AS events. The threshold for AS events with significant screening differences was a false discovery rate (FDR) of less than 0.05, while inclusion level differences greater than 5% (0.05) were considered statistically significant. Integrated Genomics Viewer (IGV, version 2.10.2) for visualization and confirmation of AS events based on RNA-seq data. Alternative 3’ splice site (A3SS) ,the 3’ end splice site is consistent but the 5’ end splice site is different, and the 5’ end exon of the second transcript is prolonged. Alternative 5’ splice site (A5SS), the 5’ end splice site is the same but the 3’ end splice site is different, and the 3’ end exon of the second transcript is prolonged. Skipped exons (SE) is when an exon is cut from the original transcript. Retained intron (RI), the second transcript is retained by retained intron to form new exons with the exons on both sides. Mutually exclusive exon (MXE), variable splicing of genes results in two different transcripts, constitutive exon which is identical and inclusive exon which is different.

### Statistical analysis

We used clusterProfile software for Kyoto Encyclopedia of Genes and Genomes (KEGG) pathway enrichment analysis of the differentially expressed genes. padj < 0.05 indicated significant enrichment. Mouse genome data (GRCm39) were used as the reference. We used GraphPad Prism software (version 7.0) to analyse the statistics. Student’s t test was used for this study. Data are expressed as the means ± SEMs in at least three independent experiments. *P* < 0.05 was considered statistically significant. * *P* < 0.05, ** *P* < 0. 01, *** *P* < 0.001, **** *P* < 0.0001.

## Results

### The expression of BCAS2 in the mouse ovary

**Fig. 1:**
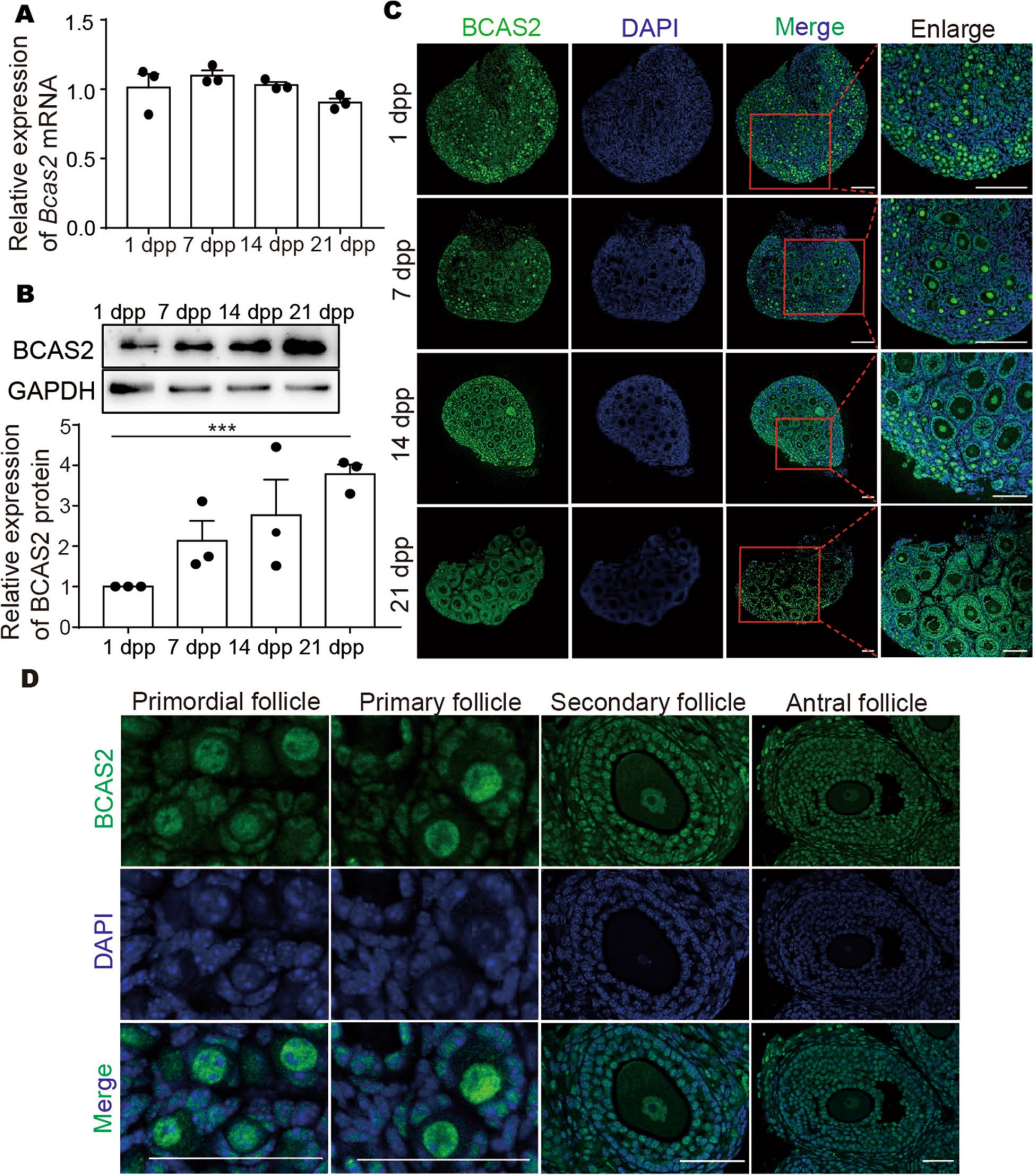
BCAS2 expression pattern during follicle development.** A** Relative *Bcas2* mRNA expression levels in ovaries on different days. The results are presented as the means ± SEMs. Three independent experiments were included for each data point. **B** The BCAS2 protein levels were detected on different days by Western blot. The results are presented as the means ± SEMs. Three independent experiments were included for each data point. *** *P* < 0.001. **C** Localization of BCAS2 in 1 dpp, 7 dpp, 14 dpp, and 21 dpp ovaries by immunofluorescence. Scale bars, 100 μm. **D** Localization of BCAS2 in follicles at various developmental stages. Scale bars, 50 μm

The expression of BCAS2 was detected in ovaries on different days by qPCR, Western blot, and immunofluorescence. qPCR results revealed that *Bcas2* did not have a significant change at the mRNA level at 1 dpp, 7 dpp, 14 dpp, or 21 dpp (Fig. 1A). However, Western blot results showed that BCAS2 expression increased from 1 dpp to 21 dpp (Fig. 1B). Immunofluorescence results showed that BCAS2 was located in the nucleus of both oocytes and granulosa cells at 1 dpp, 7 dpp, 14 dpp, and 21 dpp (Fig. 1C). Furthermore, BCAS2 is localized in the granulosa cells and oocytes of primordial follicles, primary follicles, secondary follicles, and antral follicles (Fig. 1D).

## *Bcas2* was successfully knocked out in granulosa cells

**Fig. 2:**
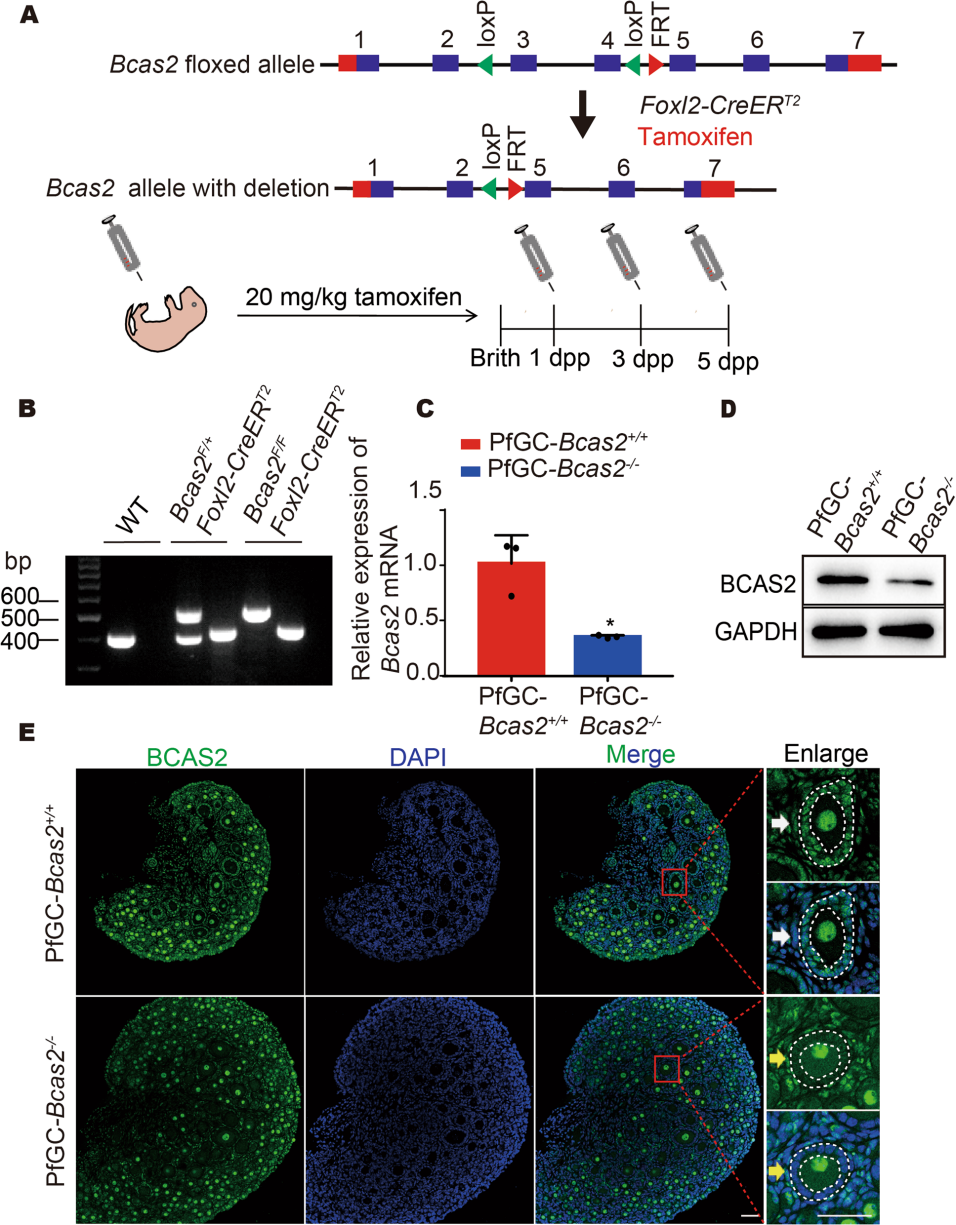
*Bcas2* knockout in granulosa cells at 7 dpp ovaries. **A** Schematic illustration of deletion of *Bcas2* exons by injecting tamoxifen. **B** The WT, *Bcas2*^*F/+*^; *Foxl2-CreER*^*T2*^, and *Bcas2*^*F/F*^; *Foxl2-CreER*^*T2*^ genotypes were detected by common PCR. **C, D** Real-time PCR and Western blot assays of the knockout efficiency of *Bcas2* at 7 dpp. T tests were performed to compare each group, * *P* < 0.05. Each test had at least three independent replications. **E** Expression of BCAS2 in the PfGC-*Bcas2*^*+/+*^ and PfGC*-Bcas2*^*−/−*^ ovaries. The areas circled by the dotted white lines represent granulosa cells. The white arrow indicates PfGC-*Bcas2*^*+/+*^ granulosa cells, and the yellow arrow indicates PfGC*-Bcas2*^*−/−*^ granulosa cells. Scale bars, 50 μm

The granulosa cell *Bcas2*-cKO mice were generated by crossing *Bcas2*^*F/F*^ mice with loxP sites flanking exons 3 to 4 with *Foxl2-CreER*^*T2*^ mice. Mice were injected with 20 mg/kg tamoxifen at 1 dpp, 3 dpp, and 5 dpp to induce *Bcas2* deletion in granulosa cells, which we refer to as primordial follicle granulosa cells (pfGCs) at these stages [30] (Fig. 2A). For the convenience of subsequent description, *Bcas2*^*F/F*^ called PfGC-*Bcas2*^*+/+*^, *Bcas2*^*F/F*^; *Foxl2-CreER*^*T2*^ called PfGC*-Bcas2*^*−/−*^. PCR was used to detect genotypes (Fig. 2B). Real-time PCR showed that *Bcas2* was dramatically decreased at the mRNA level in the 7 dpp PfGC-*Bcas2*^*−/−*^ ovary (Fig. 2C). Western blot confirmed that BCAS2 was significantly reduced at the protein level at 7 dpp in the PfGC*-Bcas2*^*−/−*^ ovaries (Fig. 2D). Immunofluorescence showed that BCAS2 was specifically deleted in the granulosa cells of primary follicles at 7 dpp (Fig. 2E). However, BCAS2 was still detectable in pfGCs, and the probable reason may be that the accumulation of BCAS2 protein in PfGCs was not completely degraded (Fig. S1C). And tested the specificity of the antibody by immunofluorescence and Western blotting and the results showed the specificity of BCAS2 antibody (Fig. S1D, E).

### Follicle development was arrested in the primary stage in PfGC-*Bcas2*^-/-^ ovaries

**Fig. 3:**
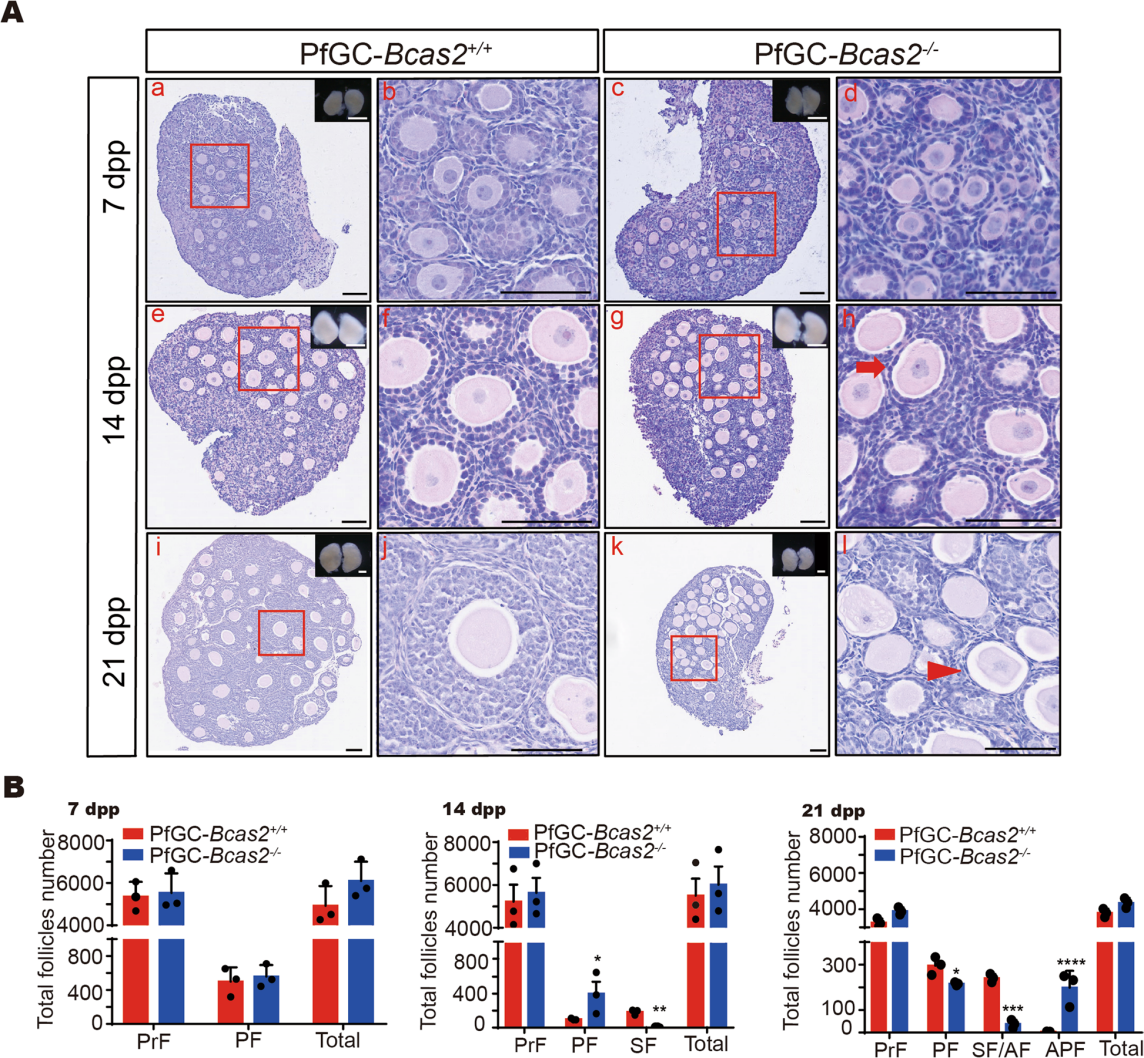
Deletion of *Bcas2* in granulosa cells impairs ovary development. **A** H&E staining of 7 dpp, 14 dpp, and 21 dpp ovaries from the PfGC-*Bcas2*^*−/−*^ and PfGC*-Bcas2*^*−/−*^ mice. The arrow indicates a primary follicle with an enlarged oocyte in PfGC-*Bcas2*^*−/−*^ mice. The arrowhead indicates an atretic follicle in PfGC-*Bcas2*^*−/−*^ mice. Black scale bars, 100 μm; white scale bars, 500 μm. **B** Quantitative analysis of follicle types from 7 dpp, 14 dpp, and 21 dpp mice in the PfGC-*Bcas2*^*+/+*^ and PfGC*-Bcas2*^*−/−*^ ovaries. PrF, primordial follicles; PF, primary follicles; SF, secondary follicles; AF, antral follicles; APF, atretic follicles. Student’s t test was used for this study. Data are expressed as the means ± SEMs in at least three independent experiments. * *P* < 0.05, ** *P* < 0. 01, *** *P* < 0.001, **** *P* < 0.0001

To explore the function of BCAS2 in granulosa cells, we analysed ovarian histology on different days by H&E staining. At 7 dpp, histological analyses revealed that the PfGC-*Bcas2*^*+/+*^ and PfGC*-Bcas2*^*−/−*^ ovaries contained primordial follicles and primary follicles (Fig. 3A-b, d), and there were no significant size differences in ovaries (Fig. 3A-a, c). Follicle counting results showed that no significant differences were found in follicle numbers between the PfGC-*Bcas2*^*+/+*^ and PfGC*-Bcas2*^*−/−*^ groups (Fig. 3B). To clarify whether primordial follicle activation is altered by the loss of BCAS2 in granulosa cells, 7 dpp ovaries were stained for FoxO3a and DDX4, the results showed that FoxO3a was located in the nucleus and cytoplasm in the PfGC-*Bcas2*^*+/+*^ and PfGC*-Bcas2*^*−/−*^ oocytes (Fig. S1A). There was no significant difference in the proportion of FoxO3a in the cytoplasm between the PfGC-*Bcas2*^*+/+*^ and PfGC*-Bcas2*^*−/−*^ oocytes (Fig. S1B). The reason may be that BCAS2 is still present in the pfGCs (Fig. S1C). At 14 dpp, an apparent morphological difference was observed between the PfGC-*Bcas2*^*+/+*^ and PfGC*-Bcas2*^*−/−*^ groups. The PfGC-*Bcas2*^*+/+*^ ovaries were larger than the PfGC*-Bcas2*^*−/−*^ ovaries because the PfGC-*Bcas2*^*+/+*^ ovaries already contain secondary follicles (Fig. 3A-f); however, in the PfGC*-Bcas2*^*−/−*^ ovaries, there were only primary follicles with an enlarged oocyte (Fig. 3A-h), due to continuous growth of the oocytes, and proliferation arrest of granulosa cells, primary follicles in PfGC-*Bcas2*^*−/−*^ ovaries could be larger than that of PfGC-*Bcas2*^*+/+*^. At 21 dpp, the PfGC-*Bcas2*^*+/+*^ ovaries were much larger than the PfGC*-Bcas2*^*−/−*^ ovaries (Fig. 3A-i, k) because PfGC-*Bcas2*^*+/+*^ follicles had more secondary follicles and antral follicles (Fig. 3A-j), which had multiple granulosa cell layers; however, few secondary follicles and a large number of atretic follicles were observed in the PfGC*-Bcas2*^*−/−*^ ovaries (Fig. 3A-l and B). Thus, these results suggest that primary follicles cannot develop beyond secondary follicles and granulosa cells undergo apoptosis in the PfGC*-Bcas2*^*−/−*^ ovaries.

### Loss of BCAS2 repressed the G1/S transition in granulosa cells

**Fig. 4:**
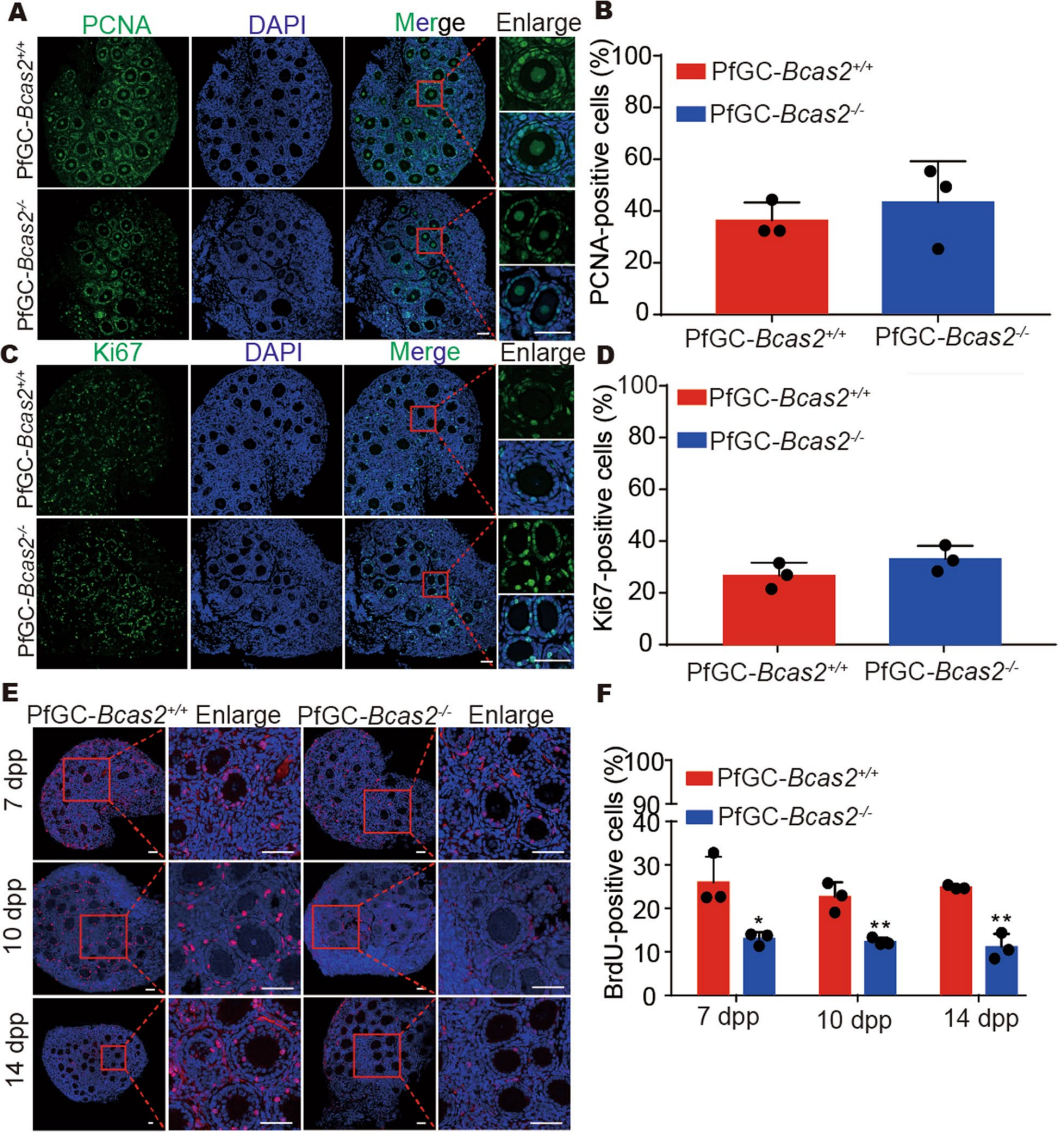
Loss of BCAS2 results in decreased BrdU^+^cells in granulosa cells.** A** The expression of PCNA in 10 dpp ovaries by immunofluorescence. Scale bars, 50 μm. **B** The percentage of PCNA^+^ in granulosa cells. Student’s t test was used for this study. Data are expressed as the means ± SEMs in at least three independent experiments. * *P* < 0.05, ** *P* < 0. 01, *** *P* < 0.001. **C** The expression of Ki67 in 10 dpp ovaries by immunofluorescence. Scale bars, 50 μm. **D** The percentage of Ki67^+^ granulosa cells. Student’s t test was used for this study. Data are expressed as the means ± SEMs in at least three independent experiments. * *P* < 0.05, ** *P* < 0. 01, *** *P* < 0.001. **E** Immunofluorescence staining of BrdU in the ovaries of the PfGC-*Bcas2*^*+/+*^ and PfGC-*Bcas2*^*−/−*^ mice at 7 dpp, 10 dpp, and 14 dpp. Scale bars, 50 μm. **F** The percentage of BrdU^+^ granulosa cells of the PfGC-*Bcas2*^*+/+*^ and PfGC-*Bcas2*^*−/−*^ mice at 7 dpp, 10 dpp, and 14 dpp. Student’s t test was used for this study. Data are expressed as the means ± SEMs in at least three independent experiments. * *P* < 0.05, ** *P* < 0. 01, *** *P* < 0.001

The absence of secondary follicles in the PfGC*-Bcas2*^*−/−*^ mouse ovaries indicates that the cell cycle of granulosa cells may be arrested. To investigate this, we analysed the expression of PCNA and Ki67 by immunofluorescence at 10 dpp (Fig. 4A, C). Cell counting results showed that there were no significant changes in the PCNA immunoreactive (PCNA^+^) cell ratio or Ki67- immunoreactive (Ki67^+^) cell ratio (Fig. 4B, D), suggesting that PfGC*-Bcas2*^*−/−*^ granulosa cells can enter the cell cycle. However, immunofluorescence and count results showed that the BrdU immunoreactive (BrdU^+^) cell rate significantly decreased at 7 dpp, 10 dpp, and 14 dpp in the PfGC*-Bcas2*^*−/−*^ granulosa cells (Fig. 4E, F). Thus, the loss of BCAS2 leads to the granulosa cell arrest in the G1 phase.

## BCAS2 deletion caused granulosa cell DNA damage and apoptosis

**Fig. 5:**
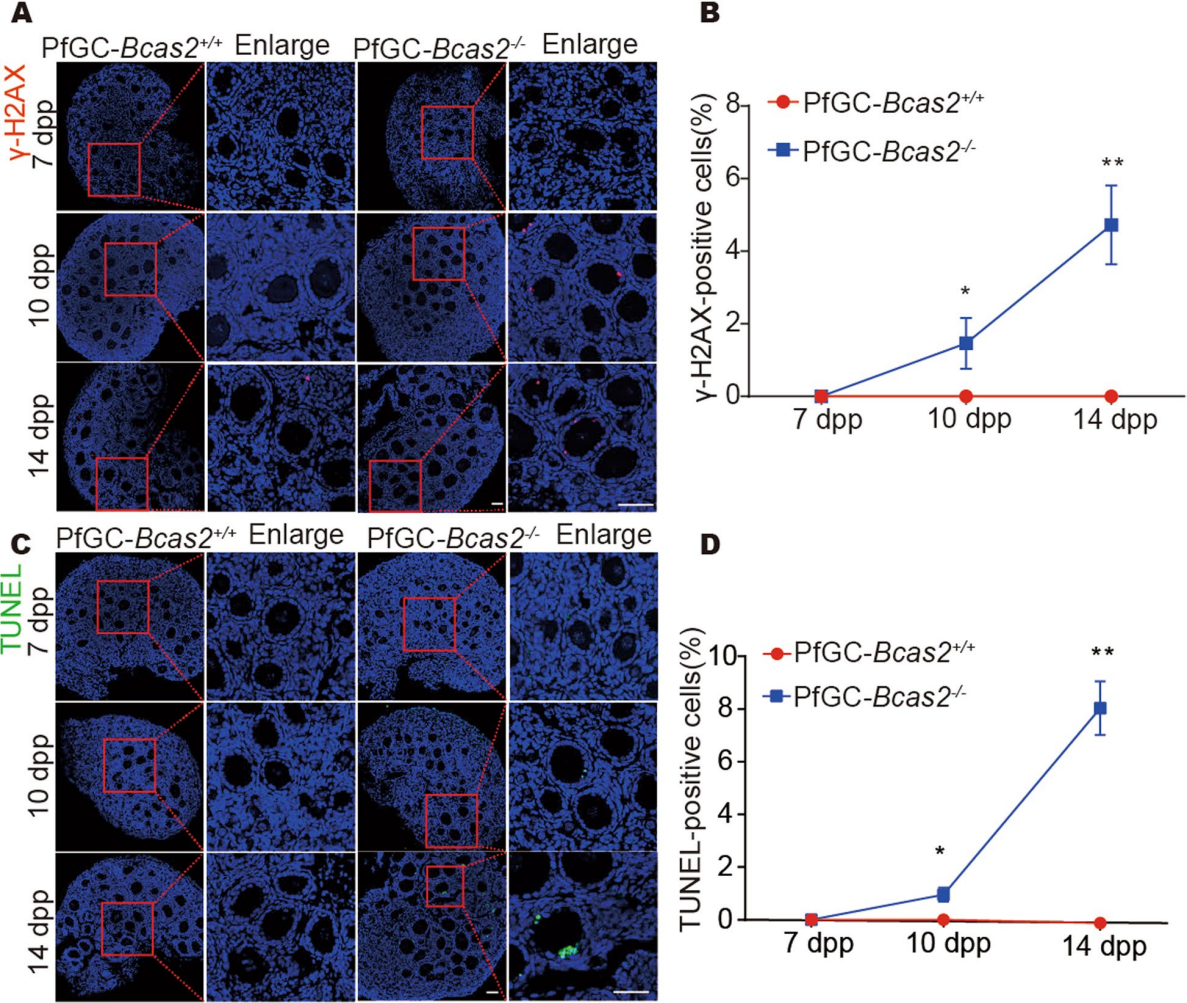
Loss of BCAS2 in granulosa cells leads to DNA damage and increased apoptosis. **A**,** C** Images of PfGC-*Bcas2*^+/+^ and PfGC-*Bcas2*^*−/−*^ ovaries were obtained at 7 dpp, 10 dpp, and 14 dpp and stained with anti-γ-H2AX and TUNEL. Scale bars, 50 μm. **B**, **D** The positive cell rate of γ-H2AX and TUNEL was counted at different times. Student’s t test was used for this study. Data are expressed as the means ± SEMs in at least three independent experiments. * *P* < 0.05, ** *P* < 0. 01, *** *P* < 0.001

To investigate whether DNA damage occurs in granulosa cells, we detected the DNA damage marker γ-H2AX using immunofluorescence. The results showed that no positive cells were observed in granulosa cells of the PfGC-*Bcas2*^*+/+*^ mouse ovaries. Whereas the γ-H2AX-positive signal appeared in granulosa cells in PfGC*-Bcas2*^*−/−*^ at 10 dpp, γ-H2AX-positive cells increased significantly at 14 dpp (Fig. 5A, B). Given that BCAS2 mutants are unable to bind RPA1, resulting in failed DNA repair at the zygotic stage [22], we next examined RPA1 staining in the PfGC*-Bcas2*^*−/−*^ and PfGC-*Bcas2*^*+/+*^ ovaries. Intriguingly, we found that the RPA1 foci in granulosa cells were significantly increased with the deletion of *Bcas2* (Fig. S2). Furthermore, TUNEL results showed that there were no positive cells in the PfGC-*Bcas2*^*+/+*^ granulosa cells at 7 dpp, 10 dpp, and 14 dpp. However, cell apoptosis increased in granulosa cells from 10 dpp to 14 dpp in PfGC*-Bcas2*^*−/−*^ ovaries (Fig. 5C, D). These results suggest that BCAS2 ablation induces DNA damage and cell apoptosis in granulosa cells.

## BCAS2 regulated the cellular senescence of functional genes

**Fig. 6:**
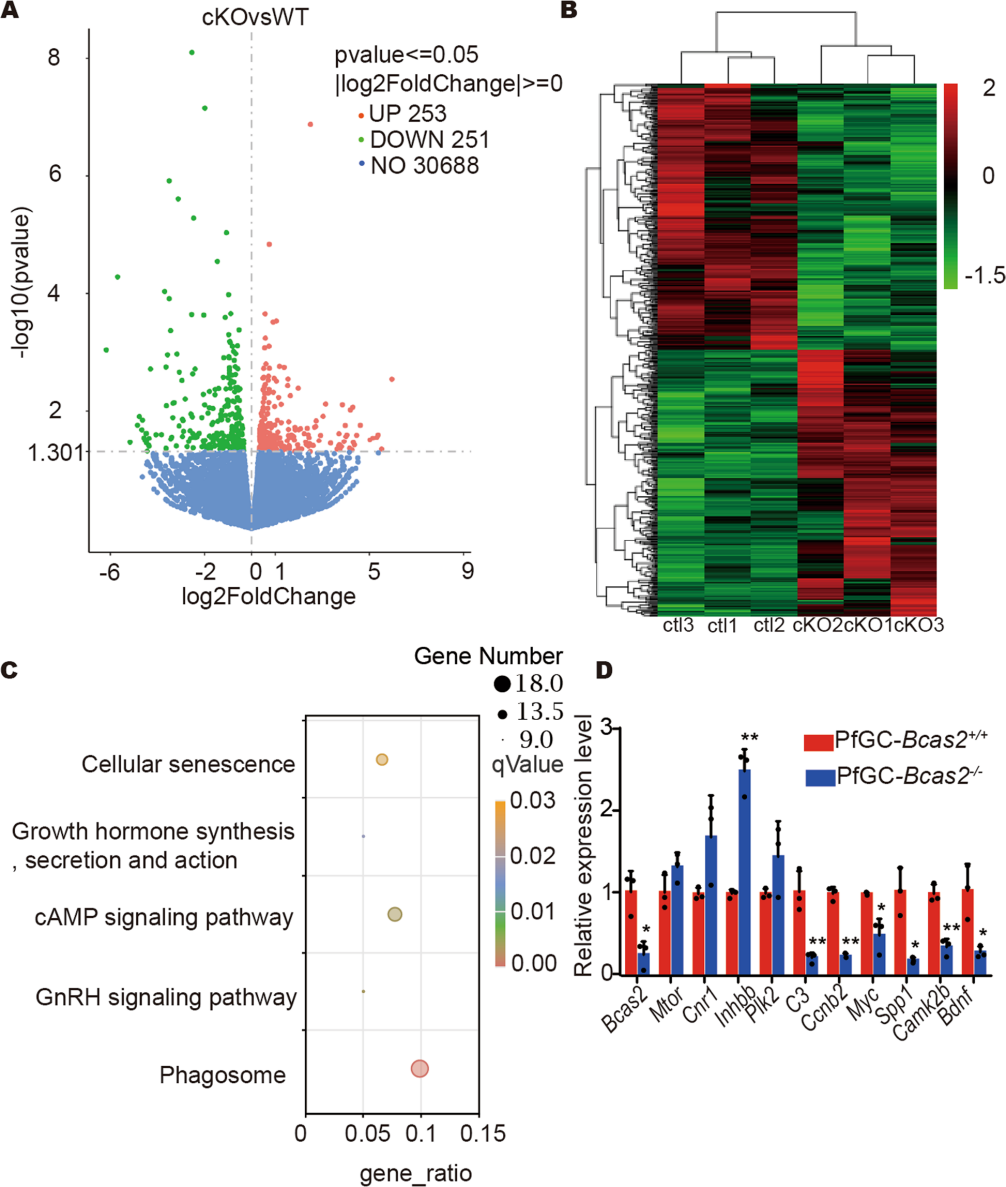
Loss of BCAS2 in granulosa cells leads to changes in cell cycle, apoptosis-related mRNA.** A** Volcano map of differentially expressed transcripts in 8 dpp PfGC-*Bcas2*^+/+^ and PfGC-*Bcas2*^*−/−*^ ovaries. Green dots are downregulated transcripts, red dots are upregulated transcripts, and blue dots are unchanged transcripts (*p*value ≤ 0.05, |log2FoldChange|≥0). **B** Cluster heatmap. Hierarchical clustering was used to cluster the FPKM values of genes, and rows were homogenized (Z score). **C** KEGG dot analysis of the significantly affected transcripts. **D** Real-time PCR analysis of the differentially expressed genes. Student’s t test was used for this study. Data are expressed as the means ± SEMs in at least three independent experiments. * *P* < 0.05, ** *P* < 0. 01

To further explore the molecular consequences of BCAS2 depletion in granulosa cells, we performed a transcriptome analysis of ovaries at 8 dpp. RNA-seq results showed that 504 genes were differentially expressed, with 253 upregulated and 251 downregulated in the PfGC-*Bcas2*^*−/−*^ ovaries (Fig. 6A, B). Kyoto Encyclopedia of Genes and Genomes(KEGG)enrichment analysis revealed that some changes occurred in cellular senescence after *Bcas2* deletion (Fig. 6C). The accuracy of the sequencing data was confirmed by real-time PCR of mRNA levels for the genes related to the cell cycle and cell apoptosis (Fig. 6D).

### BCAS2 indirectly regulated the AS of cell cycle-related transcripts

**Fig. 7:**
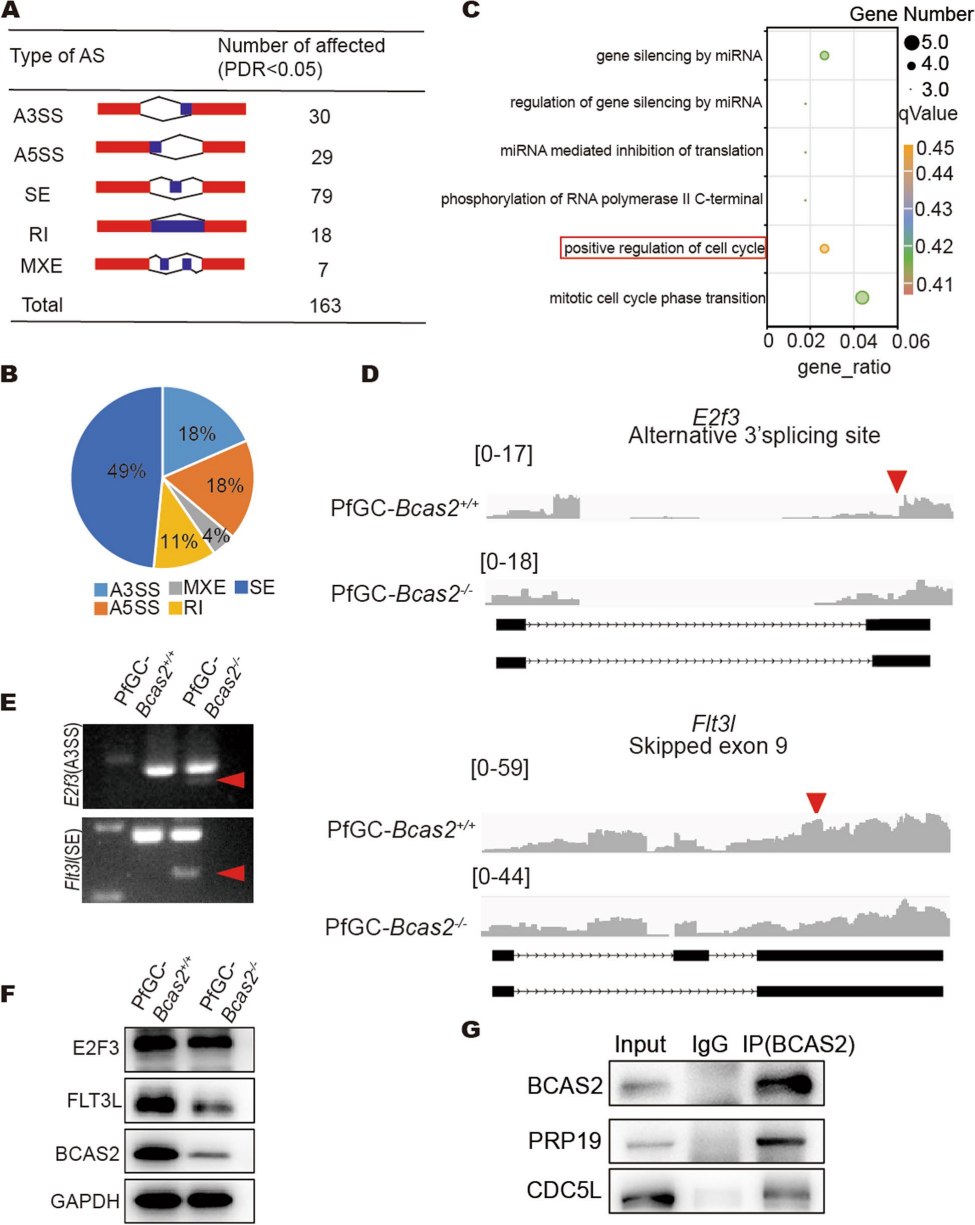
BCAS2 participates in AS by forming the PRP19 complex.** A** Five AS events significantly affected by BCAS2-deficient granulosa cells at 8 dpp. rMATS (3.2.5) software was used to analyse the AS (PDR < 0.5). **B** Pie chart was used to show the proportion of different AS types. **C** GO enrichment analysis scatter plot from the 163 affected AS events. **D** IGV visualization software shows *E2f3* and *Flt3l* AS. **E** RT‒PCR verification of AS in PfGC-*Bcas2*^+/+^ and PfGC-*Bcas2*^*−/−*^. Primers were synthesized by Sangon Biotech (Supplementary Table 4). Arrowheads indicate abnormal AS. **F** Western blot of E2F3 and FLT3L expression at 8 dpp in the PfGC-*Bcas2*^+/+^ and PfGC-*Bcas2*^*−/−*^ ovaries. **G** Co-IP analysis of BCAS2 interacts with PRP19 and CDC5L

We analysed the AS data and found that 163 genes had undergone abnormal AS in the PfGC-*Bcas2*^*−/−*^ ovaries, 30 splicing events were categorized as A3SS, 29 splicing events were categorized as A5SS, 79 splicing events were categorized as SE, 18 splicing events were categorized as RI, 7 splicing events were categorized as MXE (Fig. 7A), SE accounted for the highest proportion (Fig. 7B). The GO enrichment analysis of AS revealed that eight cell cycle-related genes showed alterations in AS forms (Fig. 7C), and we selected two of them to verify variable splicing. The variable splicing forms of the *E2f3* and *Flt3l* genes were analyzed using IGV visualization software. The results showed that *E2f3* has an A3SS AS form, and *Flt3l* has an SE AS form (Fig. 7D). RT‒PCR results show that BCAS2 regulates the AS of *E2f3* and *Flt3l* pre-mRNA in ovary (Fig. 7E). Then, we used Western blot to verify the expression of FLT3L and E2F3 at the protein level, and the results showed that E2F3 did not change significantly at the protein level; however, the protein level of FLT3L decreased significantly in the PfGC-*Bcas2*^*−/−*^ ovaries compared to that in the PfGC-*Bcas2*^*+/+*^ ovaries (Fig. 7F). RIP assays revealed that the BCAS2 antibody did not enrich the mRNA of *E2f3* and *Flt3l* in the 8 dpp ovary (Fig. S3A, B), indicating that BCAS2 is indirectly involved in AS.

According to previous reports, BCAS2 is involved in mRNA splicing through the PRP19 complex [14, 26]. To further explore how BCAS2 participates in RNA processing in granulosa cells, we performed Co-IP and revealed that BCAS2 interacts with PRP19 complex core proteins (Fig. 7G). The results showed that the protein expression levels of CDC5L and PRP19 in the PfGC-*Bcas2*^*−/−*^ ovaries decreased significantly (Fig. S3C). Immunofluorescence results revealed that PRP19 and CDC5L in granulosa cell staining decreased or even disappeared completely in granulosa cells of the PfGC-*Bcas2*^*−/−*^ ovaries (Fig. S3D). The results showed that BCAS2 regulates pre-mRNA splicing through the PRP19 complex in mouse granulosa cells and that BCAS2 is required for the stabilization of the core components of the PRP19 complex in granulosa cells.

### *BCAS2* knockdown in the KGN cell line affected cell proliferation

To investigate the function of BCAS2 in human granulosa cells, we first detected the expression of BCAS2 in the KGN cell line, and immunofluorescence results showed that BCAS2 was expressed in the nucleus of the KGN cell line (Fig. S4A). We successfully knocked down *BCAS2* in the KGN cell line using sh-RNA interference, and the sh-163 knockdown efficiency was the best (Fig. S4B). Proliferation analysis showed that the BrdU^+^ cells of sh-163 group was significantly decreased (Fig. S4C), indicating that the results of the KGN cell line are consistent with the phenotype of *Bcas2* knockout mice, which provides us with a new way to explore related human diseases.

## Discussion

BCAS2 is a pre-mRNA processing factor and has been reported as a component of the spliceosome [14, 17, 18], takes part in DNA damage repair [15, 21, 22, 24, 25] and is an estrogen receptor alpha interacting protein [31]. In mouse oocytes, a lack of BCAS2 affected the function of RPA in DNA damage repair in mouse zygotes and resulted in infertility [22]. Previous studies have shown that BCAS2 is essential for pre-mRNA splicing in spermatogonia and oocytes [25, 26]. In this study, we found that BCAS2 is expressed in oocytes and granulosa cells in all types of follicles. We deleted *Bcas2* in granulosa cells from primary follicles by *Foxl2-CreER*^*T2*^ mice. The ovaries of PfGC-*Bcas2*^*−/−*^ mice exhibited a lack of secondary follicles at 14 dpp and 21 dpp, whereas the follicles of PfGC-*Bcas2*^+/+^ ovaries developed to the antral follicle stage. Additionally, the numbers of secondary and antral follicles were significantly reduced in the ovaries of the PfGC-*Bcas2*^*−/−*^mice. Our study suggested that BCAS2-defficient granulosa cells undergo proliferation arrest and fail to differentiate into functional cells which leads to the phenotype of POI oocyte retardation. Therefore, the lack of BCAS2 in granulosa cells results in arrested follicle development.

PCNA and Ki67 are markers of cell proliferation that are expressed in the G1, S, G2, and M phases. Here, we show that PCNA^+^ and Ki67^+^ were not significantly changed at 10 dpp, by which we cannot judge the proliferative arrest state of granulosa cells after *Bcas2* deletion. Hence, by incorporating BrdU, we demonstrated that the loss of BCAS2 in granulosa cells significantly reduced the number of S-phase cells in granulosa cells, suggesting that BCAS2 plays an important role in regulating the G1/S transition of the cell cycle. These results are similar to previous results showing that BCAS2 is involved in controlling *Drosophila* cell growth [14] and regulating the proliferation of prostate cancer cells [15, 32].

Cell cycle arrest is constantly accompanied by DNA damage and apoptosis [33]. DNA damage prevents cells from entering the cell cycle. S phase is a particularly vulnerable time for DNA damage exposure [34]. Histone variant H2AX phosphorylated on serine 139 (γH2AX) is a marker of DNA damage [35]. We found that γ-H2AX was remarkably upregulated in granulosa cells from 10 dpp to 14 dpp in PfGC-*Bcas2*^*−/−*^ mouse ovaries. This finding indicates that DNA damage cannot be repaired in BCAS2-null granulosa cells. Moreover, RPA1 is abnormally stained in PfGC-*Bcas2*^*−/−*^ granulosa cells. Furthermore, the TUNEL signal increased from 10 dpp to 14 dpp in granulosa cells.

RNA-seq results showed that deficiency of BCAS2 in granulosa cells leads to abnormal pre-mRNA splicing of some genes associated with the cell cycle, such as *E2f3* and *Flt3l*. E2F3 is a family of transcription factors that regulate cellular proliferation and differentiation [36–39]. FLT3L regulates the cell cycle in a variety of cell types. FLT3L mutations confer enhanced proliferation and survival properties to multipotent progenitors in a murine model of chronic myelomonocytic leukaemia [40]. Our data showed that FLT3L protein significantly decreased in PfGC-*Bcas2*^*−/−*^ mouse ovaries, which may contribute to cell cycle arrest, abnormal DNA repair, and apoptosis in granulosa cells. The probable reason why E2F3 does not change at the protein level is that there is variable splicing of E2F3 in BCAS2-null granulosa cells, and it is not significant enough to cause protein level changes. Thus, BCAS2-null in granulosa cells causes disordered *E2f3* and *Flt3l* mRNA splicing and leads to cell cycle arrest.

**Fig. 8:**
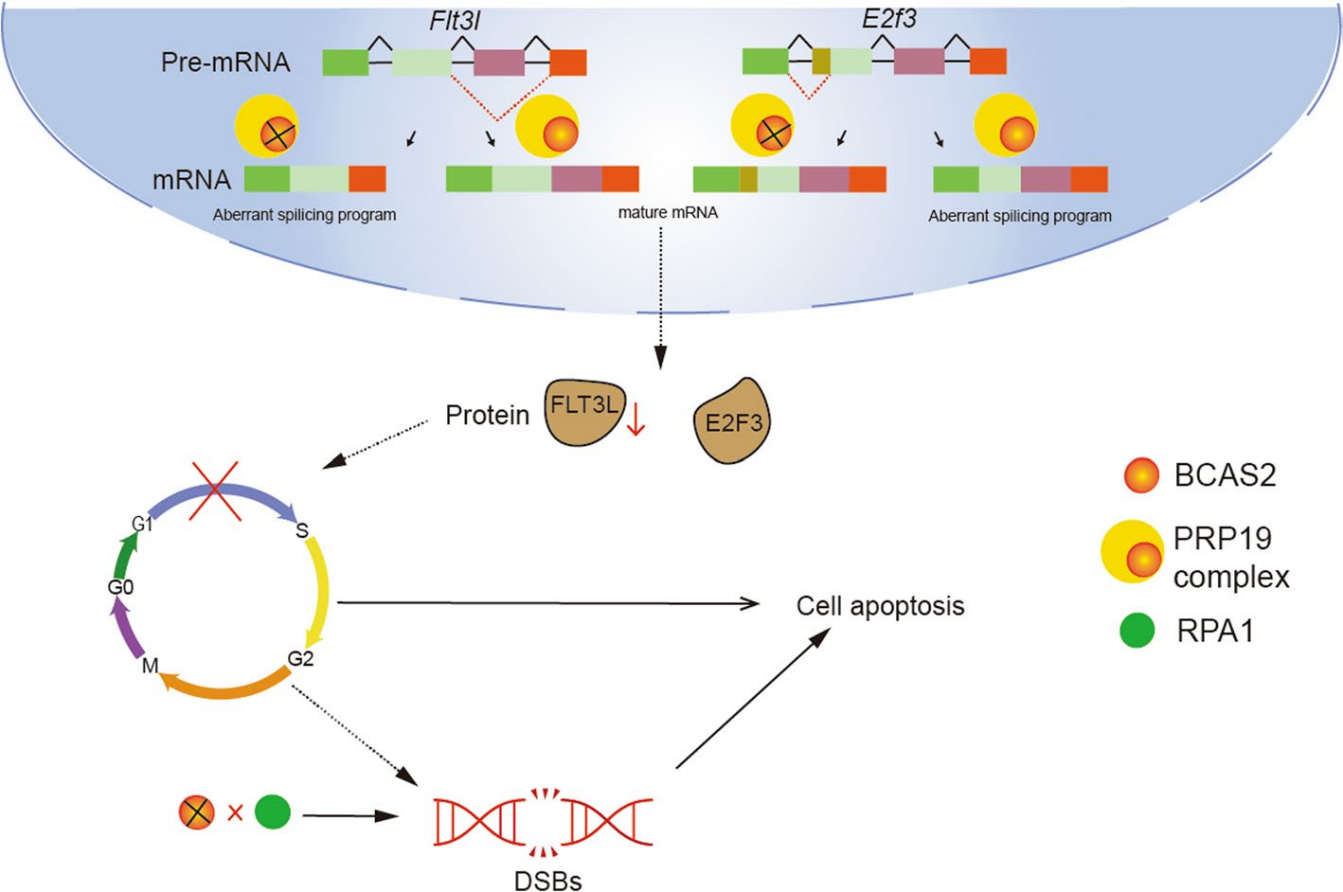
Working model for the role of BCAS2 during granulosa cell growth. BCAS2 may regulate pre-mRNA splicing of *E2f3* and *Flt3l* by forming the splicing complex with CDC5L and PRP19, controlling the proliferation and survival of granulosa cells

RIP results showed that BCAS2 antibody could not enrich *E2f3* and *Flt3l mRNA*, indicating that BCAS2 regulates AS of *E2f3* and *Flt3l* pre-mRNA through an indirect effect. Given that BCAS2 is an important component of the spliceosome, CDC5L, PRP19 and PLRG1 were examined. PRP19 is a multifunctional protein complex that mediates pre-mRNA splicing and DNA damage responses [19]. In addition to PRP19, it includes a large, highly dynamic complex consisting of U1, U2, U4/U6 and U5 small nuclear ribonucleoproteins (snRNPs) and non-snRNP proteins that undergo splicing through two successive transesterification reactions [41, 42]. The PRP19 complex is required for stable binding of U5 and U6 to the spliceosome [43]. Co-IP results showed that BCAS2 directly interacted with CDC5L and PRP19, and CDC5L and PRP19 were simultaneously significantly knockout in granulosa cells. These results indicate that BCAS2 may regulate pre-mRNA splicing of *E2f3* and *Flt3l* by forming the splicing complex with CDC5L and PRP19, thus controlling the proliferation and DNA damage of granulosa cells (Fig. 8).

In summary, our collective findings revealed that BCAS2 is essential for the proliferation and survival of granulosa cells that may be involved in regulating pre-mRNA splicing of *E2f3* and *Flt3l* as a component of the spliceosome. These findings provide new ideas for exploring granulosa cell abnormalities and potential therapeutic targets in patients with POI.

## Supplementary Information

**Additional file 1. **

**Additional file 2.**

## Data Availability

The authors confirm that the data supporting the findings of this study are available within the article and its supplementary materials. The RNA-seq data be deposited in GEO(https://www.ncbi.nlm.nih.gov/geo/query/acc.cgi?acc=GSE218919)under accession number GSE218919.
