# Supplementary material for: BCAS2 regulates granulosa cell survival by participating in mRNA alternative splicing
Source: J Ovarian Res. 2023 May 29;16:104. doi: 10.1186/s13048-023-01187-1 (PMC10226250; doi:10.1186/s13048-023-01187-1)
Supplement: Supplementary file 1 — Additional file 1. [file 13048_2023_1187_MOESM1_ESM.docx]

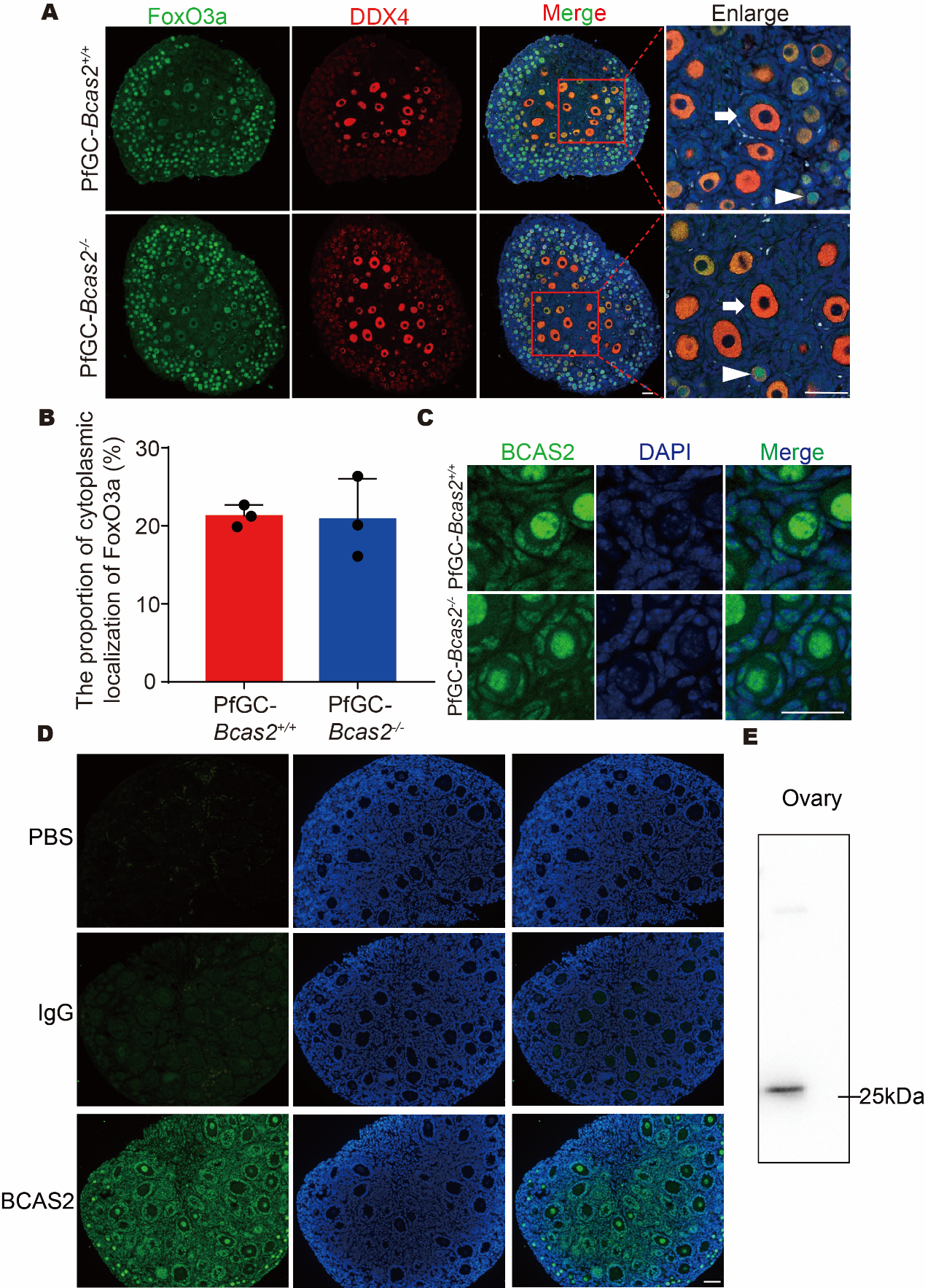


**Fig. S1 The activation of primordial follicles is normal. A** FoxO3a location in the nucleus (arrowheads) and cytoplasm (arrows) in PfGC-*Bcas2^+/+^* and PfGC*-Bcas2^-/-^* oocytes at 7 dpp. Scale bars, 50 μm. **B** The percentage of activated follicles in 7 dpp ovaries. Student’s t-test was used for this study. Data are expressed as the means ± SEMs in at least three independent experiments. * *P* < 0.05. **C** The expression of BCAS2 in primordial follicles of PfGC-*Bcas2^+/+^* and PfGC*-Bcas2^-/-^* ovary. Scale bars, 25 μm. **D** BCAS2 antibody, rabbit IgG and PBS as primary antibodies for immunofluorescence staining. Scale bars, 50 μm. **E** Mouse ovarian tissue was used as a sample for BCAS2 Western blot and the whole membrane was applied.


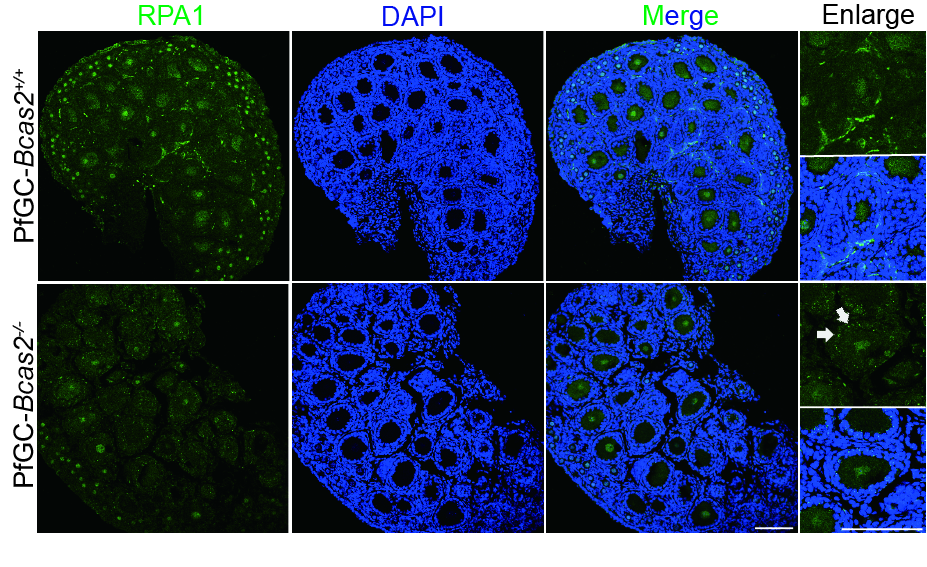


**Fig. S2 The expression of RPA1 in the ovary.** The expression of RPA1 in 14 dpp ovaries by immunofluorescence. White arrow represents positive staining. Scale bars, 100 μm.

**
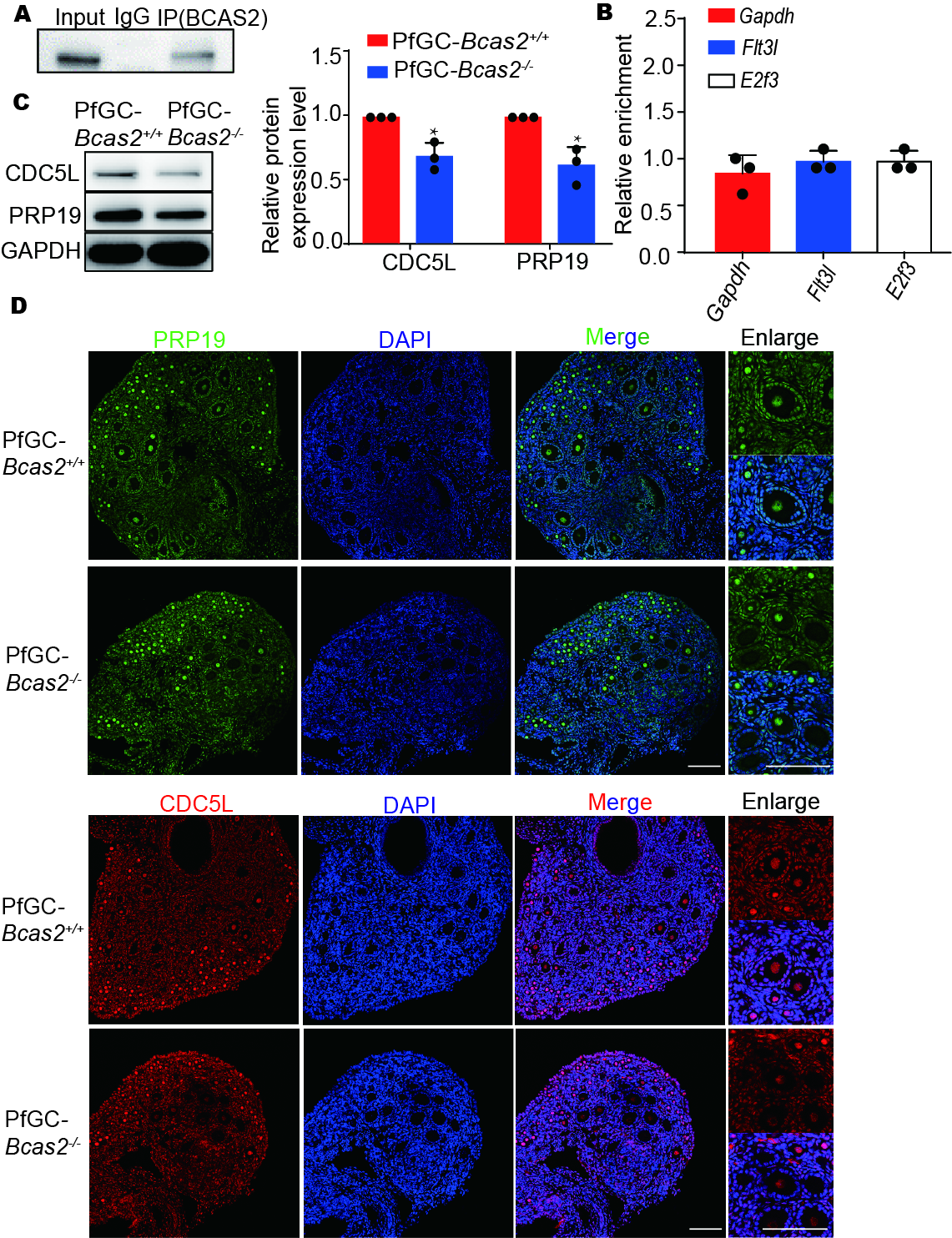
**

**Fig. S3 Decrease of the PRP19 core component in PfGC-*Bcas2^-/-^* mouse granulosa cells. A** Western blot analysis showed that BCAS2 was successfully pulled down. **B** RIP-qPCR analysis of selected genes in wild-type ovaries from 8 dpp ovaries. Means ± SEMs. Student’s t test was used for this study. Each test contained at least three independent replications. **C** Western blot analysis of CDC5L and PRP19 protein in PfGC-*Bcas2*^+/+^ and PfGC-*Bcas2^-/-^* ovaries from 8 dpp females. Student’s t test was used for this study. Data are expressed as the means ± SEMs in at least three independent experiments. * *P* < 0.05. **D** The staining of PRP19 and CDC5L in 8 dpp ovaries from PfGC-*Bcas2*^+/+^ and PfGC-*Bcas2^-/-^*. Scale bar, 100 µm.


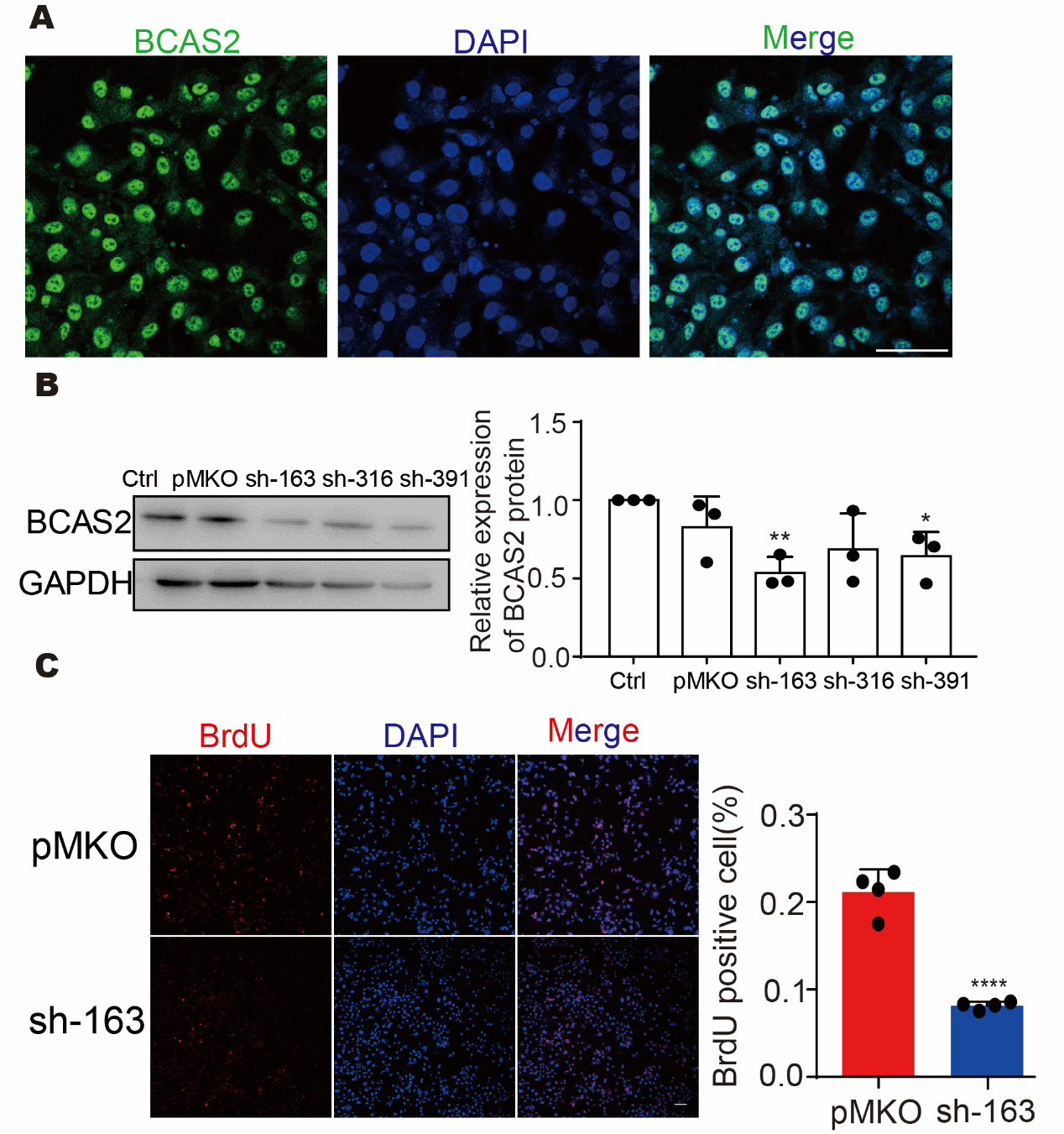


**Fig. S4 Knockdown of BCAS2 affects proliferation in the KGN cell line. A** Localization of BCAS2 in the KGN cell line by immunofluorescence. Scale bars, 50 μm. **B** Western blot assay of the knockout efficiency of BCAS2 in the KGN cell line. Student’s t test was used for this study. Data are expressed as the means ± SEMs in at least three independent experiments. **C** The percentage of BrdU^+^ in the KGN cell line. Scale bars, 50 μm. Student’s t test was used for this study. Data are expressed as the means ± SEMs in at least three independent experiments. * *P* < 0.05, ** *P* < 0. 01, **** *P* < 0.0001.
