# Supplementary material for: BCAS2 regulates granulosa cell survival by participating in mRNA alternative splicing
Source: J Ovarian Res. 2023 May 29;16:104. doi: 10.1186/s13048-023-01187-1 (PMC10226250; doi:10.1186/s13048-023-01187-1)
Supplement: Supplementary file 2 — Additional file 2. [file 13048_2023_1187_MOESM2_ESM.docx]

**Tables**

**Supplementary Table 1. Sequences of primers used for genotype identification.**

| **Genes** | **Sequences** |
| --- | --- |
| ***Bcas2* F** | **ATTCCAGCAGTTGGTGTGGG** |
| ***Bcas2* R** | **CATTGCTGGACAGAAGGTGAG** |
| ***Foxl2-CreER^T2^* F** | **ATGGACATGTTCAGGGATCG** |
| ***Foxl2-CreER^T2^* R** | **AGGGTGTTATAAGCAATCCCCAG** |

**Supplementary Table2.Antibodies used in this study**

| **ANTIBODIES** | **SOURCE** | **IDENTIFIER** |
| --- | --- | --- |
| **Rabbit anti-BCAS2 (IF, 1:100, WB, 1:1000)** | **Proteintech** | **10414-1-AP** |
| **Rabbit anti-BCAS2** | **Abmart** | **PA2466** |
| **Mouse anti-GAPDH (WB, 1:2000)** | **Proteintech** | **60004-1-IG** |
| **Rabbit anti-Ki67 (IF, 1:200)** | **Cell Signaling Technology** | **9129S** |
| **Mouse anti-DDX4 (IF, 1:400)** | **Abcam** | **ab27591** |
| **Rabbit anti-FoxO3a (IF, 1:100)** | **Cell Signaling Technology** | **12829S** |
| **anti-γ-H2AX (AlexaFluor®555)(IF, 1:500)** | **Sigma** | **05-636-AF555** |
| **Mouse anti-BrdU (IF, 1:200)** | **Developmental Studies Hybridoma Bank** | **AB 2314035** |
| **Mouse anti-PCNA (IF, 1:100)** | **Zhongshan Golden Bridge Biotechnology** | **ZM-0213** |
| **Rabbit anti-IgG (IF, 1:100)** | **Beyotime Biotechnology** | **A0181** |
| **Mouse anti-E2F3 (WB, 1:1000)** | **Santa Cruz Biotechnology** | **sc-56665** |
| **Mouse anti-FLT3L (WB, 1:1000)** | **Santa Cruz Biotechnology** | **sc-365266** |
| **Rabbit anti-RPA1 (IF, 1:500)** | **Luo LAB** |  |
| **Rabbit anti-PRP19 (IF, 1:100, WB, 1:1000)** | **Abcam** | **ab27692** |
| **Mouse anti-CDC5L (IF, 1:100, WB, 1:1000)** | **BD Transduction Laboratories** | **612362** |
| **Rabbit anti-P53(WB, 1:1000)** | **Proteintech** | **60283-2-Ig** |
| **GAR-488 (IF, 1:500)** | **Invitrogen** | **35552** |
| **GAM-594 (IF, 1:500)** | **Invitrogen** | **35510** |
| **GAM-FITC (IF, 1:50)** | **Zhongshan Golden Bridge Biotechnology** | **ZF-0312** |
| **GAR-HRP (WB, 1:8000)** | **Zhongshan Golden Bridge Biotechnology** | **ZDR-5306** |
| **GAM-HRP (WB, 1:8000)** | **Zhongshan Golden Bridge Biotechnology** | **ZDR-5307** |
| **IP™ HRP, Goat Anti-Mouse IgG LCS(WB, 1:1000)** | **AmyJet Scientific** | **AMJ-AB2016** |

**Supplementary Table 3. Sequences of primers used for qPCR.**

| **Genes** | **Sequences** |
| --- | --- |
| ***Bcas2* F** | **ACATGCACAGAAAGAGCTTCAG** |
| ***Bcas2* R** | **CGACACCCAGTTTGACTCCA** |
| ***Mtor* F** | **TTCCTGAACAGCGAGCACAA** |
| ***Mtor* R** | **GTAGCGGATATCAGGGTCAGG** |
| ***Cnr1* F** | **TCCGCCTCCTTCTGGCTC** |
| ***Cnr1* R** | **CCAGGAGGGAACCCCTTCG** |
| ***Inhbb* F** | **CAGCTTTGCAGAGACAGATGGC** |
| ***Inhbb* R** | **GTCTCCGTGACCCTGTTCTT** |
| ***Plk2* F** | **GAACCTCATGGATGGTGGTGA** |
| ***Plk2* R** | **CACCTGAAATGTGCCGTCAT** |
| ***C3* F** | **CCCCTTACCCCTTCATTCCTT** |
| ***C3* R** | **AGCCGTAGGACATTGGGAGTA** |
| ***Myc* F** | **GTTGGAAACCCCGCAGACAG** |
| ***Myc* R** | **ATAGGGCTGTACGGAGTCGT** |
| ***Spp1* F** | **GCAGCTCAGAGGAGAAGAAGC** |
| ***Spp1* R** | **TTCTGTGGCGCAAGGAGATT** |
| ***Camk2b* F** | **AGATGGAGTCAAGCCCCAGA** |
| ***Camk2b* R** | **GTGTTGGTGCTGTCGGAAGA** |
| ***Bdnf* F** | **TGCGGATATTGCGAAGGGTTA** |
| ***Bdnf* R** | **ACCTGGTGGAACATTGTGG** |

**Supplementary Table 4. Sequences of primers used for RT-PCR**

| **Genes** | **Sequences** |
| --- | --- |
| ***Flt3l* F** | **TTCAGCCACAGTCCCATCTC** |
| ***Flt3l* R** | **CCCTGCCACAGTCTTCAGTT** |
| ***E2f3* F** | **AAAGACCCCCAAGGGCAAAG** |
| ***E2f3* R** | **ACCTCTGCTGCCTTGTTCAG** |

**Supplementary Table 5. Abbreviations**

| **Abbreviations** | **Meaning** |
| --- | --- |
| **BCAS2** | **Breast cancer amplified sequence 2** |
| **AS** | **Alternative splicing** |
| **KGN** | **Human granulosa-like tumour** |
| **POI** | **Primary ovarian insufficiency** |
| **POF** | **Premature ovarian failure** |
| **PCOS** | **Polycystic ovary syndrome** |
| **RPA** | **Replication protein A** |
| **Dazl** | **Deleted azoospermia-like** |
| **Co-IP** | **Coimmunoprecipitation** |
| **RIP** | **RNA immunoprecipitation** |
| **pfGCs** | **Primordial follicle granulosa cells** |
